# Association of Racial Disparity of Cannabis Possession Arrests Among Adults and Youths With Statewide Cannabis Decriminalization and Legalization

**DOI:** 10.1001/jamahealthforum.2021.3435

**Published:** 2021-10-29

**Authors:** Brynn E. Sheehan, Richard A. Grucza, Andrew D. Plunk

**Affiliations:** 1 Department of Psychiatry and Behavioral Sciences, Eastern Virginia Medical School, Norfolk; 2 Healthcare Analytics and Delivery Science Institute, Eastern Virginia Medical School, Norfolk; 3 Department of Family and Community Medicine, Saint Louis University, St Louis, Missouri; 4 Department of Health Outcomes Research, Saint Louis University, St Louis, Missouri; 5 Department of Pediatrics, Eastern Virginia Medical School, Norfolk

## Abstract

**Importance:**

Despite calls for cannabis decriminalization and legalization, research investigating the association of policy with arrest rates and racial disparities is scarce.

**Objectives:**

To examine racial differences in cannabis arrest rates among adults and youths after statewide decriminalization, legalization, and no policy changes.

**Design, Setting, and Participants:**

This case-control study used race-based arrest data from the Uniform Crime Reporting Program and Surveillance, Epidemiology, and End Results county-level population data from January 2000 through December 2019. Data were analyzed in July 2021. Event-study analyses based on the arrest rates from 43 US states were conducted to compare preimplementation and postimplementation differences in arrest rates for states with decriminalization, legalization, and no policy changes.

**Exposures:**

Nine states implemented legalization, 8 implemented decriminalization, and 26 had no policy change.

**Main Outcomes and Measures:**

Outcome measures were cannabis arrest rates for Black and White adults and youths per year and by state, while controlling for several covariates.

**Results:**

Rates were reported per 100 000. When comparing absolute differences in arrests from January to December 2008 (before policy changes) to January to December 2019, legalization was associated with 561 and 195 fewer arrests and decriminalization with 448.6 and 117.1 fewer arrests for Black and White adults, respectively. States without a policy change saw reductions of 47.5 and 33.0 arrests for Black and White adults, respectively. Among youth, legalization was associated with 131.1 and 131.2 fewer arrests and decriminalization with 156.1 and 124.7 fewer arrests for Black and White youths, respectively. Among states without a policy change, arrests reduced by 35 and 52.4 for Black and White youths, respectively. Plotted trends of the arrest ratios from January 2000 through December 2019 suggests racial disparities remained over time. Event-study analyses suggest that decriminalization was associated with an arrest rate reduction for Black and White adults and youths. The timing of reductions suggests differential policy effects. Arrest disparities increased in states that did not have a cannabis policy change.

**Conclusions and Relevance:**

In this case-control study of states with and without cannabis decriminalization and legalization policies, increased arrest rate disparities in states without either policy highlight the need for targeted interventions to address racial injustice.

## Key Points

**Question:**

How are statewide cannabis policies (eg, legalization, decriminalization, no policy change) associated with cannabis arrest rate racial disparities among adults and youths?

**Findings:**

In this case-control study of 43 US states with and without cannabis policy changes, decriminalization and legalization were associated with large reductions in race-based arrests among adults; however, the timing of effects suggests differential policy effects. Among youth, only decriminalization was associated with reductions in arrests and arrest disparities; cannabis arrests for adults and youth increased over time in states that did not implement a cannabis policy change.

**Meaning:**

The study findings suggest that increases in arrest rate disparities in states without legalization or decriminalization highlight the need for targeted interventions to address racial injustice.

## Teaser

This case-control study examines racial differences in cannabis arrest rates in 43 states among adults and youths after statewide decriminalization, legalization, and no policy changes.

## Introduction

A 2013 American Civil Liberties Union report^1^ identified that although Black and White individuals use cannabis at roughly the same rate, Black individuals are almost 4 times more likely to be arrested for possession. The report called for legalization as the way to combat the arrest rate disparity.^1^ Despite calls for change, research that investigates the association of policy changes with adult and youth arrest rates and racial disparities is scarce, and guidance on the most effective policy remains unclear.

There is increasing support for cannabis law reform, substantially to reduce racial arrest disparities.^2^ *Legalization* refers to policies that allow for the legal sale and consumption of recreational cannabis for individuals 21 years and older. A fundamental aspect of legalization is a legal market to facilitate sales. *Decriminalization* is a reduction or removal of criminal penalties for small amounts of cannabis that are meant for personal use, while the production and sale remain prohibited. With decriminalization, if charged with possession, individuals typically face civil penalties rather than criminal charges. Although cannabis remains an illegal substance at the federal level, as of February 2021, 15 states and Washington, DC have voted to legalize adult recreational use, and 31 states and Washington, DC have decriminalized small amounts for personal consumption.^3^

Although decriminalization and legalization appear to be associated with reduced arrest rates across racial groups, they do not affect the relative racial disparity.^4,5^ Following the legalization of recreational cannabis in Washington State, there was a marked reduction in cannabis arrest rates for Black individuals; however, the relative Black-White arrest disparity grew.^6^ Across multiple examinations, the Black population saw the largest decline in absolute arrests, and the White population saw the largest proportional decline. This suggests that policies may have no effect or potentially increase the relative racial disparity over time.^5,6,7^ Additionally, prior work has examined specific policy reforms, such as all-age decriminalization^8,9^ or legalization for individuals 21 years or older.^4,6^ Overall, decriminalization is associated with reduced possession arrests for youths and adults,^9^ and legalization is associated with reduced absolute arrest rates but not relative disparities.^6^

Most cannabis possession arrests occur among adolescents and young adults, disproportionately affecting Black boys and young men.^10^ While the American Academy of Pediatrics maintains that youths should refrain from cannabis use, they also endorse its decriminalization given the severe consequences of punishment; including jail time, monetary fines, and the long-term stigma of drug conviction, much of which is considered more harmful than the use of the drug.^11^ Despite some concerns that decriminalization might increase adolescent use, research generally does not confirm these fears.^9,12,13^ For example, while decriminalization was associated with large reductions in arrest rates for adults and youths, it was not associated with an increase in use or other rule-violating behavior among adolescents.^9^ Recently, Plunk et al^13^ examined how policies were associated with adult and youth arrest rates across 38 states. Although states that implemented legalization experienced reductions in cannabis-related arrests, this effect was found only among adults and had no association with the arrest rates of minors.

To our knowledge, no studies have compared arrest rate disparities associated with different cannabis reform policies with those in states that have not yet implemented any cannabis policy reform. In this article, we examined racial differences in adult and youth arrest rates after statewide cannabis decriminalization, legalization, and no policy changes. Our primary goal was to assess how the timing of cannabis-related policy changes are associated with differences between Black and White adult and adolescent cannabis possession arrest rates and how any changes in rates compare with states without policy changes. Using 19 years of data, this study contributes to the literature by assessing raw estimates of arrest data as well as testing rates associated with the specific timing of policy reform from 10 years before through 3 years after implementation.

## Methods

This study was determined by the Eastern Virginia Medical School institutional review board to not involve human participants; therefore, informed consent was waived. Methods and results are reported according to Strengthening the Reporting of Observational Studies in Epidemiology (STROBE) reporting guidelines.

### Source Data

Arrest data were obtained from the publicly available Uniform Crime Reporting (UCR) program data and included counts of arrests by age, sex, and race from January 2000 through December 2019.^14^ Because of race category reporting changing over time, only data for Black and White individuals were included in the study. State population estimates by race were obtained from the Surveillance, Epidemiology, and End Results (SEER) program and included intercensal bridged estimates derived from US Census data (1990-2019).^15^ Data were used to generate separate adult (age ≥18 years) and youth (age <18 years) data sets with Black and White arrest rates per year and state. The UCR data were incomplete for Illinois, Florida, and Washington, DC throughout the study period. Further, data for Colorado were incorrect because of an error in reporting for Denver, Colorado. To remedy this, we obtained correct counts of arrests directly from the Denver Police Department. We did not find other potential misreporting of data.

### Cannabis Policy Coding

Decriminalization and legalization policies were verified by examining the legislative databases for each state. Date of implementation, rather than policy passage date, was used for coding. If features of a state’s policy occurred in phases (eg, possession was legalized in Washington in December 2012, and commercial sales began in July 2014), coding was based on when the penalty changed (eg, December 2012 for Washington). Policy variables were coded as 0 or 1 except during transition years, for which fractional values were used (eg, 0.5 for a policy change during June). During the analysis period, Arkansas and North Dakota increased penalties for possession; Maine, Nebraska, and Ohio already had an aspect of cannabis decriminalization; and Kansas, Kentucky, Louisiana, Nevada, and New York decreased penalties but fell short of statewide decriminalization. These states, in addition to Illinois, Florida, and Washington, DC, for which data were incomplete, were excluded from analyses. One state, Delaware, decriminalized adult possession but kept criminal penalties for youths; as such, Delaware was considered a decriminalization state in adult analyses and a control state in youth analyses. State categorization and years of policy changes are listed in the eTable in the Supplement.

### Outcome Measures

The primary outcome measure was state cannabis possession arrest rates. Cannabis possession arrests are their own category in the UCR data, with reporting following a hierarchy rule; a multioffense incident with a part I offense is reported as the most severe crime (eg, homicide, robbery), and part II offenses (eg, cannabis possession) are reported only if they occurred without a part I offense.^16^ Thus, cannabis possession arrests in the study are nonviolent drug offenses. Arrest rates were calculated by dividing the total number of arrests for each race and age group in each state by the total population for that group and multiplying by 100 000.

### Statistical Analysis Plan

The overall goal of the case-control study was to compare preimplementation and postimplementation differences in arrest rates for states with a policy change with those without a policy change.^17,18^ All values are reported as the mean number of arrests per 100 000 persons. To understand the arrest rate changes compared with absolute arrest rates, we first reported the mean number of arrests from 2008 (before implementation of any policy) and from 2019 (the end of the observed time series). We then presented racial arrest rate ratios over time to compare the relative racial disparity between legalization, decriminalization, and no policy change states by age group. Finally, we performed event-study analyses with 10 leading and 3 lagged policy indicators.^19^ Standard errors were adjusted to allow for clustering at the state level. Unordered categorical variables for state and year were used to control for invariant state and time effects. In addition to the 2 categorical indicators for decriminalization and legalization policy, we controlled for several state-level covariates: the percentage of individuals for several age and racial and ethnic groups, median income, number of police per capita, unemployment rate, poverty rate, percentage of population with a college degree, smoke-free air policy score, presence of a medical cannabis policy, and a citizen political ideology measure.^20,21,22^ Because arrest rate data are available only in aggregate (ie, not individual level), separate models were estimated for arrest rates of Black adults, White adults, Black youths, and White youths. Version 3.4.4 of R (R Foundation) was used for all analyses.^23^

## Results

### Raw Arrest Rates

**Figure 1.**
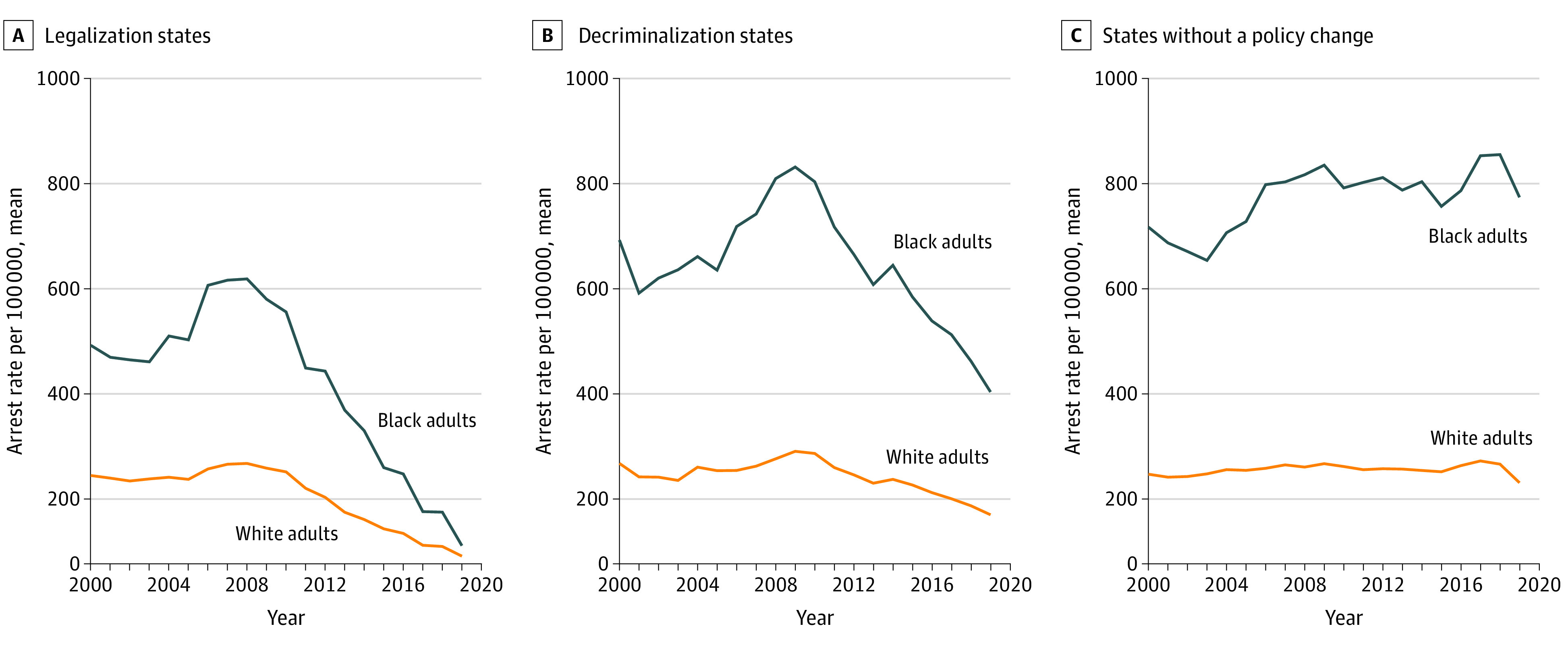
Adult Arrest Rate Trends of Cannabis Possession by Race Between Legalization, Decriminalization, and No Policy Reform States

Analyses were based on 43 states comprising 9 legalization, 8 decriminalization, and 26 control states that did not implement a cannabis policy during the study period. In states that implemented legalization, the arrest rate in 2008 was 599.2 for Black adults and 210.9 for White adults. The corresponding numbers in 2019 were 38.0 and 15.9, respectively. In states that decriminalized, the 2008 arrest rates were 810.0 and 220.0 for Black and White adults, respectively. In 2019, these rates were 361.4 and 102.9 for Black and White adults, respectively. Among states without a policy change, arrest rates increased over time for Black adults and remained stable for White adults. A slight decrease in arrests for Black and White adults was seen from 2018 to 2019. In 2008, these rates were 818.5 and 203.3 for Black and White adults, respectively; the corresponding rates in 2019 were 771.0 and 170.3. Raw arrest rates are plotted by year in Figure 1.

**Figure 2.**
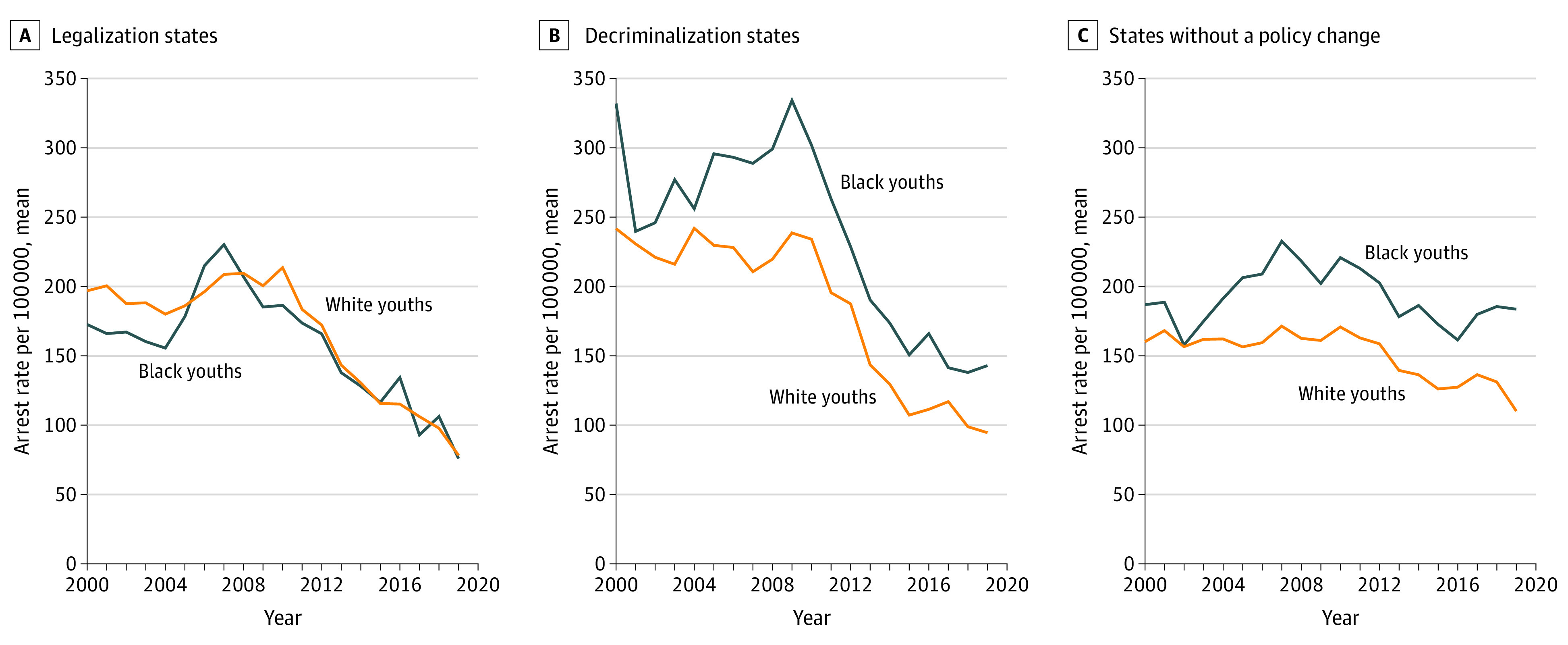
Youth Arrest Rate Trends of Cannabis Possession by Race Between Legalization, Decriminalization, and No Policy Reform States

Among youths, in 2008, before policy changes, the Black youth arrest rate was 207.0 in states that eventually legalized and 209.5 for White youths. In 2019, these rates were 75.9 and 78.3, respectively. In 2008, rates in decriminalization states were 299.1 and 219.7 for Black and White youths compared with 143.0 and 95.0, respectively, in 2019. Among states without a policy change, arrest rates for Black and White youths reduced slightly from 2008 through 2019; rates were 218.3 and 162.6, respectively, in 2008 compared with 183.3 and 110.2 in 2019 (Figure 2).

**Figure 3.**
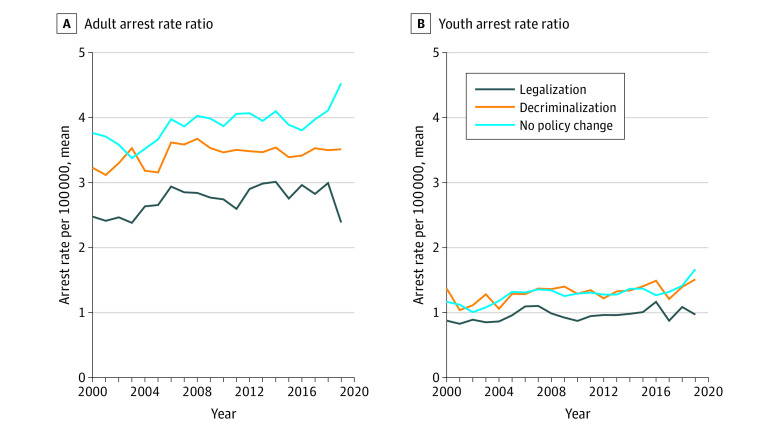
Adult and Youth Relative Racial Arrest Rate Ratio Between Legalization, Decriminalization, and No Policy Reform States

The racial arrest rate ratios from 2000 through 2019 are plotted in Figure 3. Among adults, the arrest rate ratio remained relatively constant over time. States that implemented legalization saw a reduction in the arrest disparity from 2018 to 2019, whereas states that had no policy changes saw an increase in the relative racial disparity during this period. Among youths, legalization and decriminalization were associated with a reduction in the relative racial disparity from 2016 to 2017; however, the disparity grew during the following years. Similar to the adult population, no policy change was associated with an increase in the relative racial disparity.

### Event-Study Analyses

**Figure 4.**
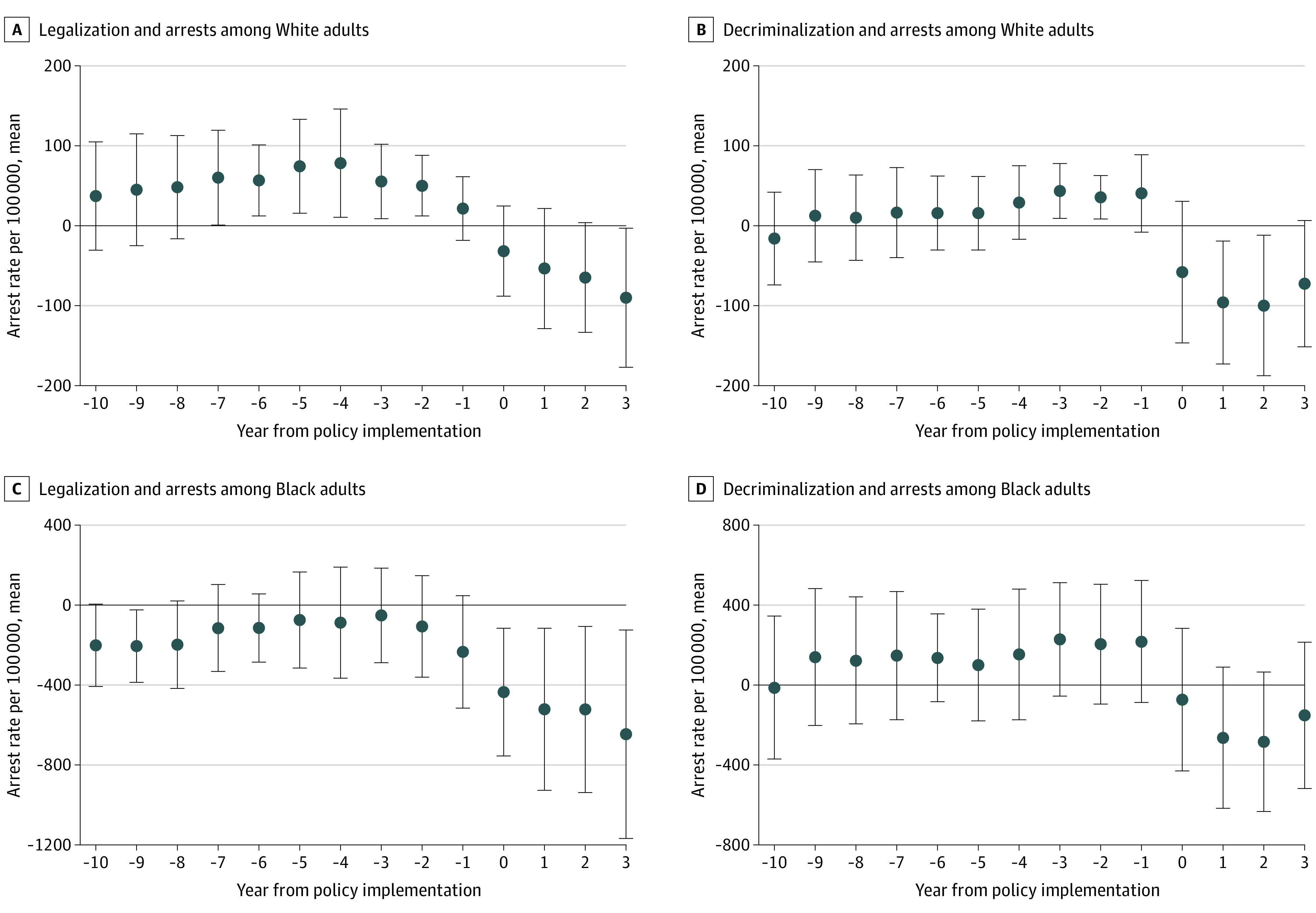
Adjusted Adult Arrest Rate Trends of Cannabis Possession by Race Before and After Cannabis Policy Change Error bars indicate 95% CIs.

**Figure 5.**
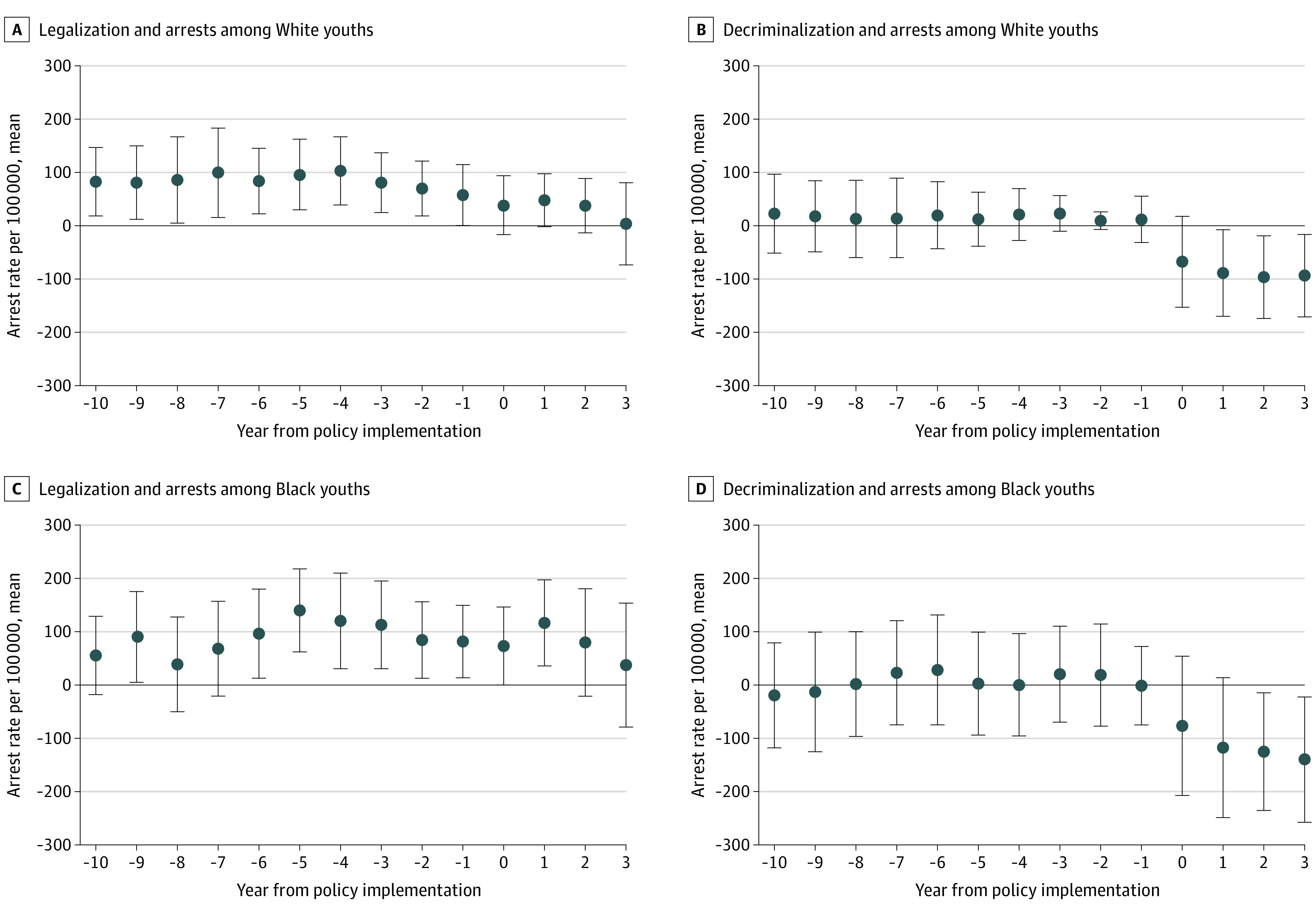
Adjusted Youth Arrest Rate Trends of Cannabis Possession by Race Before and After Cannabis Policy Change Error bars indicate 95% CIs.

Model estimates are presented in Figure 4 (adult) and Figure 5 (youth). Among adults, legalization states exhibited 1-year and 2-year anticipatory effects for Black adults and 1-year, 2-year, and 3-year anticipatory effects for White adults. In other words, arrest rates in these states were decreasing before the implementation of cannabis legalization. In decriminalization states, an arrest rate reduction was seen for Black and White adults during the year of policy implementation. Specifically, the year of decriminalization implementation was associated with an arrest rate reduction of 292 arrests for Black adults and 98 for White adults.

Among Black and White youths, legalization was associated with an increase in arrests during the year following policy implementation, which interrupted already occurring decreases in arrests. Among decriminalization states, arrest rate reductions mirrored those of adults, with relatively flat prepolicy trends followed by decreases after policy implementation. Specifically, the decriminalization implementation year was associated with an arrest rate reduction of 76 arrests for Black youths and 78 for White youths.

## Discussion

Overall, results revealed that states that implemented a cannabis policy change saw large decreases in arrests compared with states that had no policy reform. However, among adults, the association between the timing of these reductions and ongoing trends in arrest rates suggest differential policy effects and raise questions about the generalizability of these effects to other states. Specifically, while legalization appears to be associated with the largest decrease in raw differences between Black and White arrests; these reductions do not necessarily reflect the actual effect of policies because the downward trend begins well ahead of implementation. The decriminalization trend reflects a more abrupt change in absolute arrests at the time of policy implementation (Figure 4); however, the relative race-based disparity remained over time (Figure 3). Most notably, states that did not implement any policy change showed no meaningful change in arrests for White individuals and an increase for Black individuals, thereby increasing the arrest rate disparity over time. Further, findings differed for youths in that decriminalization appeared to benefit Black and White youths (Figure 5) and was associated with a notable reduction of a racial disparity in 2017; however, the relative disparity remained during the subsequent years (see raw trends [Figure 2] and arrest rate ratios [Figure 3]). Results were less stark for states with legalization, and the racial disparity remained in states without a cannabis policy change.

As illustrated in Figure 1 and Figure 4, there was a substantial decrease in absolute arrests among Black adults as an immediate response to decriminalization; however, the relative racial disparity remained. In states that adopted legalization, there was a clear reduction in arrests among Black and White adults as well as a reduction in the racial disparity from 2018 to 2019. However, as the reduction trend began before the policy implementation, this reduction was likely not solely associated with the direct effect of cannabis legalization. Results suggest that reductions in Black arrest rates began 2 years before legalization and continued to reduce during the year of policy changes (Figure 4). Arrest rates in states that implemented decriminalization did not decrease until policy implementation, suggesting a high likelihood that the reduction was directly associated with decriminalization policy. The timing of the associated differences between decriminalization and legalization is an important finding; although reductions in arrests are clear after the legalization of cannabis, other social and political factors were also likely associated with this reduction.

The arrest rate reduction timing for legalization and decriminalization has important implications for future policy implementation and efficacy. The arrest rate reduction for Black adults before legalization suggests that another common cause that could be associated with arrest rates and the decision to legalize cannabis. Social factors, such as changing social norms that drove statewide ballot initiatives and led to local jurisdictions deciding not to arrest for possession of small amounts of cannabis (effectively decriminalizing it), are likely associated with these reductions. This calls into question whether other states that might decide to implement legalization in the future will be similarly affected in terms of arrests and disparity rates unless the same social factors are taking place.

Absolute arrest trends showed little change in White and Black youth arrest rates in states that implemented cannabis legalization, which was unsurprising, considering that youths are excluded from a legalized market that targets individuals 21 years and older. Still, the need remains for targeted policies to address youth arrests and arrest disparities, as well as continued monitoring of policy effects. Further, the lack of an arrest rate reduction in legalization states emphasizes the need to attend to criminal penalties separately from addressing the question of legal markets. Black and White youths also benefited similarly from decriminalization, unlike their adult counterparts.

### Public Health Implications

The decrease in possession arrests among decriminalization states coinciding with its implementation suggests that the policy itself is accounting for the change. While states that implemented legalization were already experiencing marked reductions in arrests before the policy, states with decriminalization show evidence that the policy itself is the most salient impetus of an arrest rate reduction. For example, study results confirmed that in decriminalized states, arrests began to reduce during the implementation year and continued to reduce over time. The pattern seen in decriminalization states may represent the initial reduction that seen in legalization states before policy implementation. This similar pattern of effects raises doubt about how different these 2 policies are in practice. Both policies are ultimately followed by a similar outcome, with legalization being associated with a more robust reduction and being primed earlier on, likely by social changes. Importantly, while there were large absolute decreases in the adult arrest rate over time, particularly in states that decriminalized cannabis, relative disparities remained.

The absolute reduction in arrests among states with policy reform could have important implications for social equity. As noted, many argue that the severe consequences of possession convictions are more harmful than the health effects of cannabis use. Policy reform would not only reduce or eliminate monetary fines, but reduce court appearances, jail time, and probation, as well as the associated stigma. Further, with policy reform, steps could and should be taken to remedy cases in which individuals are currently serving time in jails or prisons because of possession arrests. In some cases, such a policy reform could expunge entire arrest records, improving, among others, recidivism rates, job placement, and housing security. Therefore, the short-term and long-term social equity effects of cannabis policy reform are widespread and multiplicative. Importantly, results suggest that these benefits will not be seen among states that do not implement any policy reform, as disparities in these states continue to increase.

Despite these likely benefits, decriminalization, which is essentially a civil penalty, may still exacerbate existing racial disparities. For example, a small fine may be merely a nuisance to those with means but serve as a burden to those without. If one of the goals of cannabis policy change is to ameliorate a racially driven policy that has advantaged some and targeted others, this is a warranted point of discussion when considering reform.

Finally, and most important concerning public health implications, arrest disparities seem to have increased over time in states that did not have a cannabis policy change. For youths, although arrest rates decreased over time for White and Black youths, the disparity remains. For adults, there was a clear increase in racial disparity as possession arrest rates remained stable for White individuals and increased consistently for Black individuals. This increase is concerning and highlights the need for immediate policy change and implementation. Although it is necessary to deliberate what type of policy is most effective, decriminalization and legalization (among adults) are followed by reduced absolute arrest rates but have a weak association with relative racial disparity. This is in stark contrast to the increase in arrests seen in states without a cannabis policy change, suggesting that adult arrest rate disparities will likely continue to increase in states absent an intentional effort to address the issue.

### Limitations

The design of the study limits causal inferences; however, analyses controlled for many potential confounders to delineate associations between policy and arrest rates and ensure strong external validity of findings. Given the secondary data analysis nature of the project, the study was limited to the quality of the arrest and statewide data available. As detailed in the Methods, data were thoroughly examined to identify any potential errors or limitations, and steps to ensure the accuracy of the data were taken. Finally, although individuals who identify as other races (eg, Latinx individuals) also face disparities in the legal system, the current study was limited to the examination of Black and White adults and youths.

## Conclusions

This study highlights the importance of statewide policies in reducing cannabis possession arrests. Among adults, a similar pattern was found in states with a policy change, with arrest reductions seen in association with a decriminalization policy and a continued downward trend seen in states that legalized cannabis. Because arrest rate reductions were occurring before policy changes in those states, there is no reason to expect cannabis legalization to have as large of an immediate effect in other states. While these results do not unambiguously favor decriminalization nor legalization, increases in arrest rate disparities in states without either policy highlight the need for targeted interventions to address racial injustice.

## Notes

Supplement. **eTable.** State data
