# Supplementary material for: Association of Racial Disparity of Cannabis Possession Arrests Among Adults and Youths With Statewide Cannabis Decriminalization and Legalization
Source: JAMA Health Forum. 2021 Oct 29;2(10):e213435. doi: 10.1001/jamahealthforum.2021.3435 (PMC8727041; doi:10.1001/jamahealthforum.2021.3435)
Supplement: Supplement. — eTable. State data [file jamahealthforum-e213435-s001.pdf]

Supplemental Online Content

Sheehan BE, Grucza RA, Plunk AD. Association of racial disparity of cannabis possession arrests among adults and youths with statewide cannabis decriminalization and legalization. *JAMA Health Forum*. 2021;2(10):e213435. doi:10.1001/jamahealthforum.2021.3435

eTable. State data

This supplemental material has been provided by the authors to give readers additional information about their work.

© 2021 Sheehan BE et al. *JAMA Health Forum*.

eTable. State data

Legalization States (year of implementation). N=9
AK (2015), CA (2017),* CO (2013), MA (2017),* ME (2017), MI (2019), OR (2015), VT (2018),* WA (2013)
**Decriminalization States (year of implementation). N=8**
CA (2011),* CT (2011), DE (2019 – adults only), MA (2009),* MD (2014), NH (2018), RI (2013), VT (2013)*
**Excluded States (reason). N=11**
AR and ND (increased penalties); ME, NE and OH (pre-existing partial decriminalization); KS, KY, LA, NV, NY (partial decriminalization during study period); IL (incomplete data).
**Control States. N=26**
AL, AZ, FL, GA, HI, IA, ID, IN, MN, MO, MS, MT, NC, NE, NJ, NM, OK, SC, SD, TN, TX, UT, VA, WI, WV, WY
*Indicates a state that implemented both types of policy but not at the same time.

© 2021 Sheehan BE et al. *JAMA Health Forum*.
